# Epithelioma of the Cervix Uteri

**Published:** 1878-01

**Authors:** Edward N. Brush


BUFFALO

### Medical and Surgical Journal.

VOL. XVII. JANUARY, 1878. No. 6.

### Original Communications.

———:o:———

ART. I.—*Epithelioma of the Cervix Uteri.* Amputation by Prof. JAMES P. WHITE, M. D.* Report of Case by EDWARD N. BRUSH, M. D.

The following case is reported for two reasons: First, it illustrates the value of operative interference, even in well advanced cases of epithelioma; and, second, as demonstrating the value of the Galvano-Cautery in certain cases.

Mrs. ———, a German woman, aged forty-nine, was referred to Prof. White for operative treatment, by Dr. Hauenstein, of this city. Her history is as follows: she is the mother of a large family; her health has always been good, and her family history shows no trace of cancer or of any diathetic malady. One of her children, an adult daughter, has well marked scrofulous cervical glands.

The patient ceased to menstruate about four years since, and the period was passed without any marked disturbance. Some four months since howerer, she began to experience pain in the back, a sense of burning accompanied by occasional lancinating pains in the uterine region and an offensive and at times copious vaginal

* This case is referred to in Trans. Buffalo Medical Association for Oct. Buffalo Medical and Surgical Journal. Vol. XVII. pp. 136.

discharge. This was briefly her condition when first seen by Dr. White. An examination revealed almost the entire posterior lip of the uterus involved in the disease, which had also made considerable inroad upon the anterior lip. The hemorrhage had been copious and frequent. The patient had become considerably reduced, and it was evident that any attempt to save her life must be immediately resorted to or it would be too late; in fact fears were expressed lest too great progress had been made by the disease. A careful examination, however, made it evident that the uterine body was not involved, and that an amputation made as near as possible at the vaginal margin would remove the entire growth. In accordance with this an operation was determined on, and on Oct. 1st the operation was performed in the presence of Drs. Hauenstein, O'Brien, Shaw, E. R. Hopkins and Brush.

Dr. Hauenstein having induced anæsthesia by means of chloroform, Dr. White siezed the uterus by a pair of strong lion forceps, and by traction aided by pressure above the pubes, brought the cervix between the labia, which were held aside by spatulæ in the hands of two assistants. A platinum loop was then thrown around the cervix and carried as high up as was deemed safe to the peritoneum, and then drawn tight. As soon as this was accomplished, a Byrne Galvano-Cautery Battery, in charge of the reporter, was attached and the current allowed to pass. As fast as the wire burned into the uterine walls it was tightened and at the same time traction was made upon the part being amputated. By this means the neck when removed had a conical shape, and the amputation was thus made at a higher point in the uterine canal than at the periphery of the neck. The amputation which was purposely made slowly, occupied but ten minutes, and was not accompanied by the slightest hemorrhage from the site of the section.

The patient was placed under my care by Dr. White and from the time of the operation until she left for home early in November not an untoward symptom presented itself. On the evening succeeding the operation the pulse was 120 temperative 100° F. which was the highest point reached by either. Anodynes were administered to relieve pain, and nutritious food and tonics freely administered. My charge of the patient terminated the first week in November, at which time the seat of the amputation had fully cicatrised and presented a smooth healthy surface both to vision and touch. The uterus was movable in the pelvis and bi-manual manipulation made with considerable pressure was unaccompanied by pain. The patient's weight had increased during the interval by some twelve to sixteen pounds, and the anxious cachectic look was fast disappearing. The condition of the patient was confirmed by Dr. White who made a careful examination about a week subsequent to my last visit. It may be said that too short a time has elapsed to report results in this case, but the result thus far has certainly justified the operation even should the disease re-appear in a month from now. The patient has been given some three months immunity from a loathsome and exhausting malady, she has increased in strength and weight and has accomplished it all at the expense of a short period of confinement to bed, which in fact was necessary before the operation, and with but little risk from the operation.

The method of operating deserves some attention. The neck of the uterus was removed entire, without loss of blood, leaving a smooth surface which on the separation of the eschar was in an excellent condition to heal by granulation. The fear of septicæmia is one which should be felt no more in the use of the cautery than with any other method, in fact Byrne who has employed it more than any American surgeon in cases of this character says that with it there is less danger from septicæmia than by other methods. The platinum loop can be placed *in situ* when cold and all of the diseased mass that is capable of removal can be embraced by it, should there remain after the major portion has been removed any portions of the cancer which can be safely taken off they can be easily amputated by the platinum knife or destroyed by the button-cautery. Such cases however offer but little inducement for operative measures as it is only when the entire mass can be removed that good results are to be expected.

